# Failure of the Anticoagulant Therapy and Psychological Distress: Still Far From a Bridge

**DOI:** 10.3389/fpsyg.2018.01709

**Published:** 2018-09-13

**Authors:** Federica Galli, Lidia Borghi, Elena Faioni, Marco Cavicchioli, Jessica Ferrari Losi, Elena Vegni

**Affiliations:** ^1^Department of Health Sciences, Università degli Studi di Milano, Milan, Italy; ^2^SIMT, ASST Santi Paolo e Carlo, Milan, Italy; ^3^Department of Psychology, Vita-Salute San Raffaele University, Milan, Italy

**Keywords:** anticoagulant therapy, failure, anxiety, depression, stress

## Abstract

**Background:** The procoagulant stress response reflects part of a beneficial adaptation of the organism to environmental threats, but a protracted procoagulant state generates a thrombotic risk. Atrial fibrillation (AF) is the most common arrhythmia in the general population. Patients with AF have a higher risk of thromboembolic events and stroke, therefore they are treated with long-term oral anticoagulant (OAC) therapy. The aim of this study is to evaluate if there is any association between psychological distress and clinically unexplained variations of the International Normalized Ratio (INR), that is the index used to monitor both thromboembolic and bleeding risk in the case of patients under OAC therapy.

**Methods:** Fifty-eight patients (men = 27; women = 31; mean age = 74.98) were recruited. The sample was divided according to the recognition (or not) of the reason why the INR was subtherapeutic (<2) and classified as “Known Reasons” (KR = 32.8%) and “Unknown Reasons” (UR = 67.2%). Psychological assessment included the following dimensions: symptoms of anxiety and depression, perceived stress, emotional regulation strategies, and alexithymia.

**Results:** Considering Mann–Whitney test results, no significant difference was found in the scores of anxiety, depression, stress, and emotional regulation strategies. With regard to alexithymia, UR patients are characterized by a moderate tendency to an outward-oriented thinking (*r* = 0.25).

**Conclusion:** A clear role for the detected psychological factors in determining abnormal INR range in patients under OAC therapy could not be found. Further studies are needed to support our findings, if possible exploring factors other than psychological distress and the related emotion regulation strategies.

## Background

Atrial fibrillation (AF) is a common disorder of cardiac rhythm (estimated prevalence in people older than 15 years in Italy is 2% (Zoni-Berisso et al., 2013) that increases with age. Psycho-emotional factors seem to be associated to AF in different ways (Galli et al., 2017). AF predisposes to arterial thrombosis and systemic embolism, the reason why patients need long-term antithrombotic prophylaxis with oral anticoagulants (OACs), as vitamin K antagonists (VKAs). Therapy with VKAs requires monitoring through a blood test, measuring the international normalized ratio (INR). In patients with AF, INR should be maintained, through dose adjustment, between 2 and 3. Lower ratios expose patients to increased risk of cardioembolism, while higher ratios to increased bleeding. Time in which the INR is in range is measured internationally by TTR (Time in Therapeutic Range). In clinical trials on anticoagulation, reported average TTR is approximately 60%, while lower levels are found in primary care; clinical studies suggest that optimal TTR should be above 70% to avoid the risk of treatment failure (Cotté et al., 2014; Pokorney et al., 2015; Vestergaard et al., 2017) Although a cause for out-of-range INR is often identified (e.g., low compliance, interference by drugs or acute intervening diseases, and long-term alcohol consumption), in approximately half of the cases cannot be found any apparent reason for the deranged INR.

Psycho-emotional reactions to stressors were found to be associated with abnormalities in coagulation (Austin et al., 2013), but little studied in patients under OAC therapy. The procoagulant stress response reflects part of a favorable adaptation of the organism to environmental threats, even if a protracted procoagulant state may generate a thrombotic risk (von Känel et al., 2005). Furthermore, “stress” was related to a fight-or-flight model to adverse health behaviors through autonomic and neuroendocrine changes, as hypercoagulability (von Känel et al., 2005). During acute mental stress, healthy individuals show a prothrombotic state that is viewed as an adaptive fight–flight response protecting the organism from excessive bleeding if injuries occur (von Känel et al., 2005). The ways these psychological characteristics may contribute or not to the INR variation from therapeutic range is little studied. A research on patients with venous thromboembolism under OAC therapy did not find any relationship between INR and negative affect (anxiety, depression, worrying, and anger), at the contrary of patients without OAC therapy (Von Känel et al., 2012).

Lagraauw et al. (2015) provided a comprehensive overview of both human and animal studies supporting the contribution of acute and chronic stress to the procoagulant state and increased cardiovascular risk Different psychological factors may determine and mediate the procoagulant state as response to emotional distress, but to the best of our knowledge they have been not yet studied in AF patients under OAC therapy (Skov et al., 2012).

Main aim of our study is to evaluate if any association exists between the unexplained failure of OAC therapy, emotional distress (anxiety/depression and perceived stress) and/or the linked emotion regulation strategies (e.g., alexithymia), with. The hypothesis is that psychological distress could have an influence on coagulation parameters leading to medication [vitamin K antagonist (VKA)] failure.

## Methods

In order to explore the role of emotional distress in patients with AF and sub-therapeutic INR, a case-control study design was used.

### Participants

The participants were drawn from the population of patients routinely followed at the anticoagulant clinic (Servizio di Immunoematologia e Medicina Trasfusionale, ASST Santi Paolo e Carlo) from January to March 2018.

Eligibility criteria for inclusion in the cohort were: (1) being patients with AF and abnormal INR (<2); (2) aged over 45 years; (3) having at least 6 months of VKA therapy (stable therapy); and (4) TTR > 65%. Exclusion criteria were: (1) a known history of cognitive impairment or cognitive decay and (2) difficulties with Italian written language.

Blood Center clinicians screened possible reasons for abnormal INR directly from the patient and assigned the patient to the group with abnormal INR for known reasons (KR) or for unknown reasons (UR). Identified reasons mean that INR sub-therapeutic value was explained by inadequate compliance, intake of drugs interfering with OAC (e.g., antibiotics), or intervening acute diseases. In the case of UR, no evident causes of abnormal INR were detected after accurate patient interview by the attending hematologist (EMF). In our OAC clinic the average TTR in recent years is 73%. Approximately a third of patients on VKAs followed at our clinic are expected to show an out-of-therapeutic-range INR at periodical monitoring 2/3 of these being subtherapeutic.

### Measures and Instruments

#### Socio-demographic and Clinical Data

Socio-demographic data included gender, age, educational level, relational status, and employment. Clinical information collected included routine assessment of health status and of medication adherence, as well as information on intervening diseases, hospital stays, and outpatient visits.

#### Anxiety and Depression Symptoms

Anxiety and depression symptoms were collected through the Italian version of the Hospital Anxiety and Depression Scale (HADS) (Costantini et al., 1999), a 14 item questionnaire addressing depressive (HAD-D) and anxious (HAD-A) symptoms, separately. The HADS can be used to determine the severity of anxiety and/or depression including absence (0–7), mild (8–10), moderate (11–14), and severe (15–21) levels (Snaith, 2003).

#### Emotion Regulation Strategies

Individual differences in the emotional regulation were evaluated using the Emotion Regulation Questionnaire (ERQ) (Gross and John, 2003; Balzarotti et al., 2010).

#### Perceived Stress

The subjective perception of stressful life events was evaluated using the Perceived Stress Scale (PSS) (Cohen et al., 1983).

#### Alexithymia

All participants completed the Toronto Alexithymia Scale (TAS-20; Bagby et al., 1994; Bressi et al., 1996 ). The TAS-20 yields three factors: DIF, difficulty in identifying feelings; DDF, difficulty in describing feelings to others; and EOT, externally oriented thinking style (Bagby et al., 1994; Bressi et al., 1996 ).

### Data Analysis

SPSS for Windows 21.0 software (SPSS Inc., Chicago, IL, United States) was used to analyze data. We controlled the comparability of KR and UR groups taking into consideration socio-demographic variables. χ^2^ test was computed in order to compare the distribution of binary variables. Conversely, given the small sample size and the violation of normality assumption even after the application of several data transformation procedures (i.e., log, square root, reciprocal, and reverse score transformations), nonparametric analyses were conducted for continuous variables. Specifically, Mann–Whitney *U* test was performed to evaluate differences between KR and UR groups considering continuous socio-demographic variables, as well as to explore the role of psychological factors on abnormal type of INR. With regard to unbalanced sample sizes of KR and UR groups, we proposed the Monte Carlo simulation using 10,000 independent samples in order to estimate the 95% confidence interval of *p*-values associated to the previous contrasts and, to assess the robustness of our findings. Moreover, *r* coefficient was used as an effect size measure of group’s comparisons (for computation procedures see: Field, 2009, p. 550). Eventually, missing data was imputed using expectation–maximization algorithm consistently with missing completely at random assumption (Little, 1988).

### Ethical Approval

The study protocol was approved by the San Paolo Hospital Ethic Committee (Comitato Interaziendale-Milano Area A). All participants gave written informed consent in accordance with the Declaration of Helsinki.

## Results

**Tables 1**, **2** show detail results from comparisons between UR and KR groups.

**Table 1:** Participants’ socio-demographic characteristics.

	Patients with unknown	Patients with known	*p-Value*
	reasons (*n* = 39)	reasons (*n* = 19)	
**Gender, % (*n*)**			0.78
Female	51.3 (20)	57.9 (11)	
Male	48.7 (19)	42.1 (8)	
**Age (years), mean (*SD*)**	75.08 (10.64)	74.78 (11.1)	0.96
Minimum age (years)	47	47	
Maximum age (years)	92	89	
**Relationship status, % (*n*)**			
Cohabiting/married	53.58 (21)	57.89 (11)	
Single/separated/widower	46.42 (18)	42.10 (8)	0.56
**Years of schooling, mean (*SD*)**	8.72 (3.49)	7.83 (2.85)	0.58
Minimum	5	5	
Maximum	13	13	

**Table 2:** Comparison between UR and KR patients among psychological variables.

	UR patients (*n* = 39)	KR patients (*n* = 19)	*U*	*Z*	*p*-Value	*r*
	Median (IQR)	Median (IQR)			(95% CI)	
**Hospital anxiety and depression scale (HADS)**						
Anxiety	5.00 (2.00-9.00)	6.00 (3.00-9.00)	354.00	−0.27	0.79 (0.78-0.80)	−0.03
Depression	5.00 (2.00-8.00)	5.00 (2.00-8.00)	368.00	−0.04	0.97 (0.96-0.98)	−0.005
**Perceived stress scale (PSS)**	15.00 (8.00-20.00)	18.50 (10.50-22.25)	288.00	−1.36	0.17 (0.16-0.18)	−0.18
**Emotion regulation questionnaire (ERQ)**						
Cognitive Reappraisal	27.00 (20.00-33.00)	29.00 (23.25-35.25)	300.00	−1.17	0.25 (0.24-0.26)	−0.15
Expressive Suppression	18.00 (14.00-20.00)	17.50 (14.50-21.25)	352.50	−0.30	0.76 (0.75-0.77)	−0.04
**Toronto alexithymia scale (TAS-20)**						
Describing	15.00 (11.00-18.00)	15.50 (12.00-17.25)	348.50	−0.36	0.72 (0.71-0.73)	−0.05
Identifying	15.26 (12.00-18.97)	17.00 (11.75-20.00)	294.50	−1.26	0.21 (0.20-0.22)	−0.16
Externally Focused	23.84 (21.00-27.00)	21.50 (17.00-24.50)	255.50	1.91	0.06 (0.05-0.07)	0.25
TAS-20 Total	53.00 (47.00-60.00)	56.50 (47.50-61.00)	351.00	−0.32	0.75 (0.74-0.76)	−0.04

UR, unknown reason and KR, known reason.

### Socio-demographic and Clinical Characteristics

Out of 72 AF patients who met study criteria, 58 agreed to participate with a response rate of 80,5%; refusing participants cited mainly time commitment required and a lack of interest in participating in this study as reasons for refusal. We found no significant differences between groups considering participants socio-demographic characteristics.

### Psychological Distress

No significant differences were observed between UR and KR individuals in scores related to HADS subscales, PSS scale, and ERQ subscales. Small to moderate difference (*r* = 0.25), although not significant, was found considering TAS-20 externally focused subscale. Specifically, UR patients showed slight higher scores than KR group.

## Discussion

Contrary to the hypothesis a clear role for the researched psychological factors in determining abnormal INR range could not be found, if not for a trend for a sub-factor of alexithymia (externally focused thinking). However, we cannot consider any significant role for this scale that is to be considered as the less reliable factor of the TAS-20 (Müller et al., 2003). In detail, we did not find a role for the recent perceived emotional distress (by the mean of PSS and HADS), in as much as both perceived stress and anxiety/depression did not discriminate the two study groups in a one-month/week time span. A likely explanation entails the absence of a role for emotional distress in determining unexplained INR variations. Similar findings had been found by a recent study on patients with myocardial infarction (Geisera et al., 2017): coagulation and fibrinolysis markers were not associated to HADS scores, but to BMI, age, gender, and smoking status. Another study on patients (with venous thromboembolism) under OAC therapy (Von Känel et al., 2012) did not find any relationship between negative affect and INR abnormal range. On the contrary, patients who had discontinued the OAC therapy showed fluctuations in INR value in the presence of psychological distress. Our findings support the result of the inconsistent burden on INR variations of the detected psychological factors, even though we cannot exclude that single coagulation factors may be influenced by emotional distress. It remains to be understood the reason why a half of patients with usual good compliance and no other factors explaining INR abnormal range (e.g., antibiotics, low compliance, interference by drugs or acute intervening diseases, and long-term alcohol consumption) shows an out of range index. Moreover, if any psychological factor influencing the INR in OAC patients exists, it should be detected in mechanisms other than recent perceived stress, alexithymia and/or psychological distress

Some shortcomings of our study need to be outlined. The number of patients included in our study may be insufficient to detect significant differences. Although we attempted to statistically control possible confounding effects of unbalanced sample sizes, future matched study are needed to definitely exclude the role of psychological factors on abnormal INR. We may suggest future research based ecological momentary assessment might capture different emotional distress trends over time considering among UR and KR groups. Additionally, it could be also possible that emotional distress is specifically implicated on the onset of abnormal INR through mechanisms other than emotional distress, alexithymia, and/or emotion regulation strategies.

Last, but not least, we cannot exclude that our psychometric tools are weak to detect the way emotional stress is handled by the elderly. In general, it is unknown whether diagnostic criteria and instruments designed for adult population are fitting for the elderly, but it is commonly believed that modifications will be necessary (Del Corno and Plotkin, 2017). The mean age of our sample is about 75 years and denotes an “old–old” population, opening to several considerations on the peculiarities of this population, such as the difficulties in recognizing important aspects of their conditions, primitive defensive patterns or accentuated depressive feelings (Del Corno and Plotkin, 2017).

In synthesis, regarding emotional distress, we did not find any factors potentially explaining INR sub-range in AF patients. Other factors should be explored in order to understand why otherwise well-adherent patients usually in-range, suddenly show INR pathological value. A role might be hypothesized for illness perception and related coping strategies, but further studies are needed to properly assess their influence.

## Author Contributions

FG role as Principal Investigator, designed the research, supervised the work, and wrote the paper. LB performed the statistical analysis, contributed to theoretical interpretation of findings and writing of the paper. EF designed the research, enrolled and selected the patients, provided the final lecture, and approved the paper. JFL collected the data and handled the database. MC made additional statistical analyses in the revision phase. EV designed the research, supervised the work, provided the final lecture, and approved the paper.

## Conflict of Interest Statement

The authors declare that the research was conducted in the absence of any commercial or financial relationships that could be construed as a potential conflict of interest.
